# Plant ionome diagnosis using sound balances: case study with mango (*Mangifera Indica*)

**DOI:** 10.3389/fpls.2013.00449

**Published:** 2013-11-12

**Authors:** Serge-Étienne Parent, Léon E. Parent, Danilo Eduardo Rozane, William Natale

**Affiliations:** ^1^Department of Soils and Agrifood Engineering, ERSAM, Université LavalQuébec, QC, Canada; ^2^Departamento de Agronomia, Universidade Estadual PaulistaRegistro, Brazil; ^3^Departamento de Solos e Adubos, Universidade Estadual PaulistaJaboticabal, Brazil

**Keywords:** plant nutrition, ionomics, crop management, mango, compositional data analysis

## Abstract

Plant ionomes and soil nutrients are commonly diagnosed in agronomy using concentration and nutrient ratio ranges. However, both diagnoses are biased by redundancy of information, subcompositional incoherence and non-normal distribution inherent to compositional data, potentially leading to conflicting results and wrong inferences. Our objective was to present an unbiased statistical approach of plant nutrient diagnosis using a balance concept and mango (*Mangifera indica*) as test crop. We collected foliar samples at flowering stage in 175 mango orchards. The ionomes comprised 11 nutrients (S, N, P, K, Ca, Mg, B, Cu, Zn, Mn, Fe). Traditional multivariate methods were found to be biased. Ionomes were thus represented by unbiased balances computed as isometric log ratios (*ilr*). Soil fertility attributes (pH and bioavailable nutrients) were transformed into balances to conduct discriminant analysis. The orchards differed more from genotype than soil nutrient signatures. A customized receiver operating characteristic (ROC) iterative procedure was developed to classify tissue ionomes between balanced/misbalanced and high/low-yielders. The ROC partitioning procedure showed that the critical Mahalanobis distance of 4.08 separating balanced from imbalanced specimens about yield cut-off of 128.5 kg fruit tree^−1^ proved to be a fairly informative test (area under curve = 0.84–0.92). The [P | N,S] and [Mn | Cu,Zn] balances were found to be potential sources of misbalance in the less productive orchards, and should thus be further investigated in field experiments. We propose using a coherent pan balance diagnostic method with median *ilr* values of top yielders centered at fulcrums of a mobile and the critical Mahalanobis distance as a guide for global nutrient balance. Nutrient concentrations in weighing pans assisted appreciating nutrients as relative shortage, adequacy or excess in balances.

## Graphical abstract

A mobile setup comprises *D*-1 balances from *D* components. Nutrients in plant tissues are diagnosed in the unbiased balance domain while their corresponding concentrations are appreciated as relative shortage, sufficiency or excess of nutrients.

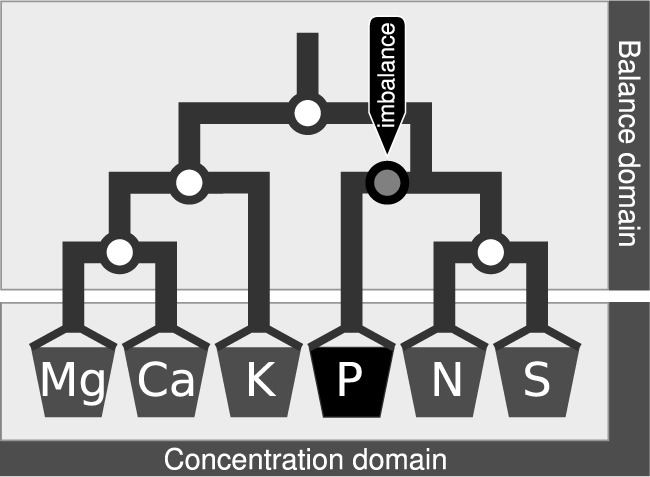



## Highlights

- Statistics on concentration data or dual ratios are biased
- Isometric log ratios (*ilr*) avoid biases due to redundancy of information, incoherence and non-normal distribution
- Foliar ionomes informatively classified orchard productivity using a novel binary classification technique based on a receiver operating characteristic technique
- The [P | N,S] and [Mn | Cu,Zn] foliar balances appeared to limit mango yields in Brazil
- The balance concept could be further developed using prior knowledge and multivariate analysis of ionomes in future studies.

## Introduction

The ionome is “the mineral nutrient and trace element composition of an organism” (Lahner et al., 2003). Nutrient concentrations included in the definition of an ionome are strictly positive data constrained between zero and the unit of measurement: they belong to the compositional data class. Consequently, each nutrient can only be analyzed relatively to the other nutrients of the ionome. Ignoring the important properties of compositional data leads to biases in their analysis due to redundancy of information, subcompositional incoherence and non-normal distribution (Bacon-Shone, 2011).

The ionome of agricultural crops is typically diagnosed using critical nutrient concentration ranges (CNCR) (Benton et al., 1991) or dual ratios possibly integrated into functions and indices by the Diagnosis and Recommendation Integrated System (DRIS) (Walworth and Sumner, 1987). The CNCR, inherited from Sprengel's “Law of minimum” stated in 1828, classifies crop nutrient as deficiency, sufficiency, luxury consumption, or excess (Epstein and Bloom, 2005). Obviously, textbooks in plant nutrition (Bergmann, 1988; Benton et al., 1991; Epstein and Bloom, 2005; Malavolta, 2006; Marschner, 2011) disregard the fundamental properties of compositional data, that have important consequences in statistical analyses.

DRIS is an empirical model (without well documented mathematical or statistical theory behind it) that computes *D* × (*D* − 1)/2 dual ratios and their associated ratio functions, then integrates functions into *D* indexes. The CNCR and DRIS are usually conducted separately, and then compared to each other to identify the most limiting nutrients (Wadt and Silva, 2010). Both CNCR and DRIS are biased and may lead to conflicting results when conducted separately (Parent et al., 2012), e.g., (Da Silva et al., 2004; Blanco--Macías et al., 2009; Huang et al., 2012; Wairegi and van Asten, 2012).

Compositional data require an appropriate transformation to avoid biases in their statistical analysis (Bacon-Shone, 2011). Aitchison (1986) proposed using the additive log ratio (*alr*) or the centered log ratio (*clr*) transformation to properly handle compositional data. The *clr* transformation was used by Parent and Dafir (1992) to rectify the DRIS by a mathematically sound model. Because a composition has rank *D*-1 (Aitchison and Greenacre, 2002), the *clr* technique, that generates *D* variables, produce a singular matrix in multivariate analysis, forcing analysts to sacrifice one *clr* variate in order to avoid singularities. Egozcue et al. (2003) proposed the isometric log-ratio transformation (*ilr*), which structures *D* components into *D*-1 orthogonal log contrasts of components amenable to multivariate analyses. In plant nutrition, these log contrasts can be defined as *ad-hoc* nutrient balances. In a functional perspective, nutrient concentrations interact (Wilkinson, 2000) within a structured system that can be partitioned into subsystems (Marschner, 2011). Such balances map the ionomes of plant species and varieties in a dimensionally reduced space (Parent et al., 2013b). Also, nutrient imbalance indexes have been computed for diagnostic purposes as a distance between an observation and the center of a reference group of balanced specimens (Parent et al., 2012).

Our objectives were (1) to present the theory of *ilr* balances for application in plant nutrition, (2) to elaborate a binary classification statistical technique to delineate a reference group and (3) to design a pan balance representation of the ionome for nutrient diagnosis using mango as test plant and (4) to demonstrate biases in traditional concentration and ratio methods used to diagnose ionomes in agronomy.

## Theory

### Compositional data

Compositional data, such as nutrient concentrations, are parts of some whole, bounded between 0 and the unit of measurement, i.e., 1, 100%, 1000 g kg^−1^, or 10^6^ mg kg^−1^. The scale of measurement is generally the dry matter mass basis (kg), but could also be the fresh matter mass basis (kg) or the sap liquid basis (L). The constrained nature of compositional data implies important properties, as follows:

- *Redundancy of information*: Where the composition is closed to one, the amount of one component can be calculated by difference between one and the sum of the others. Hence, **there are *D*-1 degrees of freedom in a *D*-parts composition**, i.e., the data set matrix has rank *D*-1 (Aitchison and Greenacre, 2002). Because any of the *D* × (*D* − 1)/2 dual ratios derivable from a *D*-parts composition can be computed from other ratios (Parent et al., 2012), they also convey redundant information that generates myriads of spurious correlations in linear statistical analysis (Chayes, 1960; Pearson, 1897).
- *Subcompositional incoherence*: **The results of statistical tests differ depending on the unit to which a composition is closed**, generating spurious correlations (Tanner, 1949). Indeed, the addition of a component such as water to the composition just provides an additional dimension to that space (e.g., a balance between water and other components). This new component should not alter the results of statistical analyses on the dry matter sub-system.
- *Non-normal distribution*: Normally distributed data are mapped in a real space, which is not the case for compositional data, which are mapped in a closed space. Statistics like confidence intervals should not be allowed to range outside the limit of the compositional space (e.g., ≤0 or ≥100%). Rather, **compositional data follow logistic-normal distributions** (Bacon-Shone, 2011).

Those three properties result from closing the compositional space, as follows (Aitchison, 1986):

(1) $$\begin{array}{l} S^{D}=C\left(c_{1},c_{2},\ldots ,c_{D}\right)\\ \;=\left(\frac{c_{1}\kappa }{\sum_{i=1}^{D}c_{i}},\frac{c_{2}\kappa }{\sum_{i=1}^{D}c_{i}},\ldots ,\frac{c_{D}\kappa }{\sum_{i=1}^{D}c_{i}}\right) \end{array}$$

where *S*^*D*^ is the simplex (compositional vector mapped in the compositional space), κ is the unit of measurement and *c*_*i*_ is the *i*th part of a composition containing *D* parts. When conducting a plant nutrient diagnosis, it is convenient to include a filling value (*Fv*) computed by difference between κ and the sum of all nutrients. Its inclusion allows back-transforming the *ilr* values into concentrations with familiar units of measurement. The main components of the filling value are C, O, and H, as found in products of photosynthesis. Lagatu and Maume (1934) were the first to apply closure to foliar nutrient data using an N-P-K ternary diagram.

### Conventional approaches

#### Nutrient concentration ranges

The CNCRs has been illustrated by the so-called Liebig's barrel filled with water, where nutrient concentrations are represented by staves of unequal length, the shortest stave being attributed to the most limiting nutrient. The rise of water in the barrel, a metaphor for plant growth, is controlled by the shortest stave. Diagnosticians who use this approach interpret concentrations as nutrient deficiency, sufficiency, luxury consumption or excess. However, such approach does not account for nutrient interactions. Concentration data are often log transformed to improve data distribution. However, the log transformation is not a panacea (Filzmoser et al., 2009), because there are still *D* variables for matrix rank of *D*-1, subcompositional incoherence is maintained, and data are constrained to the positive space.

#### Nutrient ratio ranges

Nutrient ratios have a long tradition in agronomy, aiming to capture the notion of nutrient interactions (Walworth and Sumner, 1987). Useful ratios are generally examined by data-mining, looking for high correlations with a performance index such as crop yield. However, since Pearson (1897), many statisticians (Tanner, 1949; Chayes, 1960; Aitchison, 1986) warned that the use of unstructured, correlation driven, dual ratios generate spurious correlations.

#### Nutrient stoichiometric rules

Ingestad (1987) suggested an optimum N:P:K:Ca:Mg stoichiometric rule for regulating the growth of tree seedlings, leading to ratios of all nutrients against a standard such as N, i.e., (P/N, K/N, Ca/N, and Mg/N). This approach structures ratios in a way that avoids redundancy of information and subcompositional incoherence (as long as the reference component remains in the subcomposition), but still could lead to wrong interpretations, namely due to non-normal distributions. The stoichiometric ratios could be normalized using the *alr* transformation introduced by Aitchison (1986).

#### Diagnosis and recommendation integrated system (DRIS)

The DRIS is a method to synthesize several dual ratios into nutrient indices. Dual ratios in a given ionome are first compared to DRIS dual ratio norms (ratio means and coefficients of variation obtained from high-performing specimens) to compute DRIS functions (Walworth and Sumner, 1987). The DRIS functions common to a nutrient are then added up to DRIS indices with the sign of DRIS functions depending on the position of the indexed nutrient in the ratio. Although appealing to plant diagnosticians, DRIS has poor mathematical background. Parent and Dafir (1992) rectified DRIS for plant diagnosis using the *clr* transformation introduced by Aitchison (1986).

### Compositional approaches

#### The additive and centered log ratios (alr)

The *alr* representation of compositional data is computed as follows (Aitchison, 1986):

(2) $$alr_{i}=ln\left(\frac{c_{i}}{c_{\text{common}}}\right)$$

where *c*_*i*_ is the *i*^th^ component at numerator, *i* = [1 … *D*]\*i*_common_ and *c*_common_ is the common denominator to all components, resulting in *D* − 1 *alr* values, because the component at denominator is sacrificed. Log-ratios are more tractable than ordinary ratios (Aitchison, 2003), because the inverse of a log-ratio is a trivial sign change. The *alrs* are appropriate to conduct multivariate analysis, but are not orthogonal to each other, making them difficult to interpret. The additive log ratio is a log-ratio formulation of stoichiometric rules. It should be noted that, due to their oblique geometry, distance-based statistics across additive log-ratios should be handled with care (van den Boogaart et al., 2013).

#### The centered log ratios (clr)

The *clr* representation of compositional data is computed as follows (Aitchison, 1986):

(3) $$clr_{i}=ln\left(\frac{c_{i}}{g\left(c\right)}\right)$$

where *c*_*i*_ is the *i*th component at numerator, *i* = [1 … *D*], and g(*c*) is the geometric mean of all components, resulting in *D clr* values, i.e., there is one extra degree of freedom for a matrix of rank of *D* − 1 (the *clr* variates add up to 0). One *clr* value must be sacrificed (e.g., that of the filling value) in many multivariate analyses, hence returning the adequate *D*-1 degrees of freedom, but an inappropriate geometry. Because outliers may affect considerably log ratios (Filzmoser and Gschwandtner, 2013), the diagnostic power of CND-*clr* is also decreased by large variations in nutrient levels (e.g., leaf Cu, Zn, Mn contamination by fungicides). The *clrs* are also subcompositionally incoherent. Indeed, because each component is ratioed by the geometric mean of the whole composition in Equation (3), the choice of adding or not a component (such as carbon or water content) affects the whole *clr* vector. Nevertheless, the *clr* transformation is useful to conduct exploratory biplot analyses on compositional data (Egozcue and Pawlowsky-Glahn, 2011a).

#### The isometric log ratio (ilr)

The *ilr* technique (Egozcue et al., 2003) allows projecting the simplex *S*^*D*^ of compositional data into a Euclidean space of *D*-1 non-overlapping orthogonal log contrasts, also called orthonormal balances or geometric “coordinates” (not to be confounded with spatial coordinates). A system of balances can be designed into a sequential binary partition (SBP). A SBP is a (*D* − 1) × *D* matrix, in which parts labeled “+1” (group numerator) are contrasted with parts labeled “−1” (group denominator) in each ordered row (see Table 1 for an example). A part labeled “0” is excluded from the balance. The composition is partitioned sequentially at every ordered row into two contrasts until the (+1) and (−1) subsets each contain a single part. Balances are computed as follows (Egozcue and Pawlowsky-Glahn, 2005):

(4) $$ilr_{i}=\sqrt{\frac{n_{i}^{+}n_{i}^{-}}{n_{i}^{+}+n_{i}^{-}}}ln\frac{g\text{​}\left(c_{i}^{+}\right)}{g\text{​}\left(c_{i}^{-}\right)}$$

Where, in the *i*th row of the SBP, *n*_*i*+_ and *n*_*i*−_ are the numbers of components in the plus (+) or group and the minus (−) or group, respectively, *g*(*c*_*i*+_) is the geometric mean of components in the + group and *g*(*c*_*i*−_) is the geometric mean of components in the—group. The natural log of the ratio of geometric means is a log contrast; the associated co-efficient, $\sqrt{n^{+}{}_{i}n_{i}^{-}/\left(n_{i}^{+}+n_{i}^{-}\right)}$, is an orthogonal co-efficient. The *ilr* transformation is the most appropriate for robust multivariate analysis of compositional data (Filzmoser and Hron, 2011).

**Table 1:** **Sequential orthogonal partition of eleven nutrients of plant ionome and the filling value to compute 11 *ilr* orthonormal coordinates from concentration values and orthogonal coefficients**.

**ILR ID**	**S**	**N**	**P**	**K**	**Ca**	**Mg**	**B**	**Cu**	**Zn**	**Mn**	**Fe**	***Fv***	**Notation**
1	+1	+1	+1	+1	+1	+1	+1	+1	+1	+1	+1	−1	[Fv | S,N,P,K,Ca,Mg,B,Cu,Zn,Mn,Fe]
2	+1	+1	+1	+1	+1	+1	+1	−1	−1	−1	−1	0	[Cu,Zn,Mn,Fe | S,N,P,K,Ca,Mg,B]
3	+1	+1	+1	+1	+1	+1	−1	0	0	0	0	0	[B | S,N,P,K,Ca,Mg]
4	+1	+1	+1	−1	−1	−1	0	0	0	0	0	0	[K,Ca,Mg | S,N,P]
5	+1	+1	−1	0	0	0	0	0	0	0	0	0	[P | S,N]
6	+1	−1	0	0	0	0	0	0	0	0	0	0	[N | S]
7	0	0	0	+1	−1	−1	0	0	0	0	0	0	[Ca,Mg | K]
8	0	0	0	0	+1	−1	0	0	0	0	0	0	[Mg | Ca]
9	0	0	0	0	0	0	0	+1	+1	+1	−1	0	[Fe |Cu,Zn,Mn]
10	0	0	0	0	0	0	0	+1	+1	−1	0	0	[Mn | Cu,Zn]
11	0	0	0	0	0	0	0	+1	−1	0	0	0	[Zn | Cu]

In this paper, we noted balances as “[−1 or denominator group | +1 or numerator group],” because in algebra, negative numbers are located on the left. If the minus (−1) group loads more than the plus (+1), the value of the balance is negative (i.e., leans toward the components on the left side of the vertical bar), and vice-versa.

#### Designing sequential binary partitions (SBP)

There are *D*! × (*D*-1)!/2^*D*−1^ possible different SBPs for a *D*-parts composition. In fact, the design of the SBP does not influence results in multivariate linear statistics: switching from a SBP to another is just a rotation of orthogonal axes from the origin of the coordinates of a scatter. Because information provided by an experiment depends on the prior assumption (Egozcue and Pawlowsky-Glahn, 2011b), balances can be designed as interpretable variables using prior and expert knowledge (Parent et al., 2012, 2013a,b; Aslam et al., 2013), exploratory biplot analysis of *clr* variates (Aitchison and Greenacre, 2002) and principal balances analysis (Pawlowsky-Glahn et al., 2011). In this paper, we designed balances using prior and expert knowledge only, because biplot analysis and principal balances analysis (still under development) would require exploring several datasets for nutrient relationships, which is beyond the scope of this paper.

Wilkinson (2000) listed several dual and higher-order nutrient interactions in plants in much larger numbers than the *D*-1 balances allowable from a *D*-parts composition (Aitchison and Greenacre, 2002). We thus designed a sound SBP for plant ionomes using prior knowledge on nutrient interactions (Table 1). Nutrients were first contrasted with the filling value computed by difference between unit of measurement and the sum on nutrient concentrations. Macro-nutrients and B were separated from cationic micronutrients. Macro-nutrients and B were connected, because B interacts with macronutrients (Malavolta, 2006). Macro-nutrient anions (S, N, P) were contrasted with macro-nutrient cations (K, Ca, Mg) to reflect charge balance in plant cells. Macro-nutrient anions were further subdivided according to protein synthesis (N, S) and energy (P); the [P | S,N] balance thus reflects the protein/energy relationship in plants similarly to the Redfield N/P ratio (Loladze and Elser, 2011). Macro-nutrient cations were contrasted as monovalent vs. divalent ions whereby K, Ca and Mg are competing nutrients (Marschner, 2011). Fungicides that protect agricultural plants against diseases often contain Cu, Zn, and Mn in their active molecules. The Cu and Zn were thus assumed to be mainly affected by fungicide sprays; Mn may originate from soil or fungicide sprays while soil can be assumed to be a large reservoir of Mn and Fe; the [Fe |Cu,Zn,Cu] balance is intended to reflect the effect of fungicide sprays over soil supply of cationic micronutrients. Of course, if this study had a different objective, we could, for example, have designed a SBP providing more focus on Fe:S clusters in proteins (Couturier et al., 2013).

The soil compositional data were orthogonally arranged in a SBP for the only purpose of discriminant analysis between mango genotypes.

#### Dissimilarity between two compositions

The Mahalanobis distance (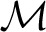) across selected *ilr* coordinates of ionomes is computed as follows:

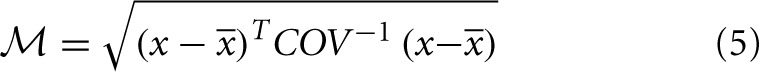



where x is the barycenter of a reference population and *COV* is the covariance matrix of the reference population. The Mahalanobis distance across *ilr* balances [values of *x* in Equation (5)] is a measure of the multivariate distance between a diagnosed and a reference composition [values of x in Equation (5)].

The Mahalanobis distance was preferred over the Euclidean distance, widely used when variables are dimensionally homogeneous and orthogonal. The Mahalanobis distance, thanks to the covariance matrix included in its definition, can account for the usual inclined hyper-ellipsoidal shape of plant ionome scatters (Parent et al., 2012; Marchand et al., 2013).

### Binary classification method

For diagnostic purposes, there is a need to split the crops into low- and high-productivity groups. A predictor index should allow separating balanced from misbalanced nutrient signatures. Four quadrants are partitioned in system diagnosis (Swets, 1988), where each quadrant delineates a response class (Table 2) according to response and predictor delimiters. The Mahalanobis distance is computed from the center and the co-variance of the reference population. To define an optimal predictor delimiter, Nelson and Anderson (1977) proposed to maximize the “Class sum of squares” between two groups clustered by the predictor delimiter for a given response delimiter.

**Table 2:**
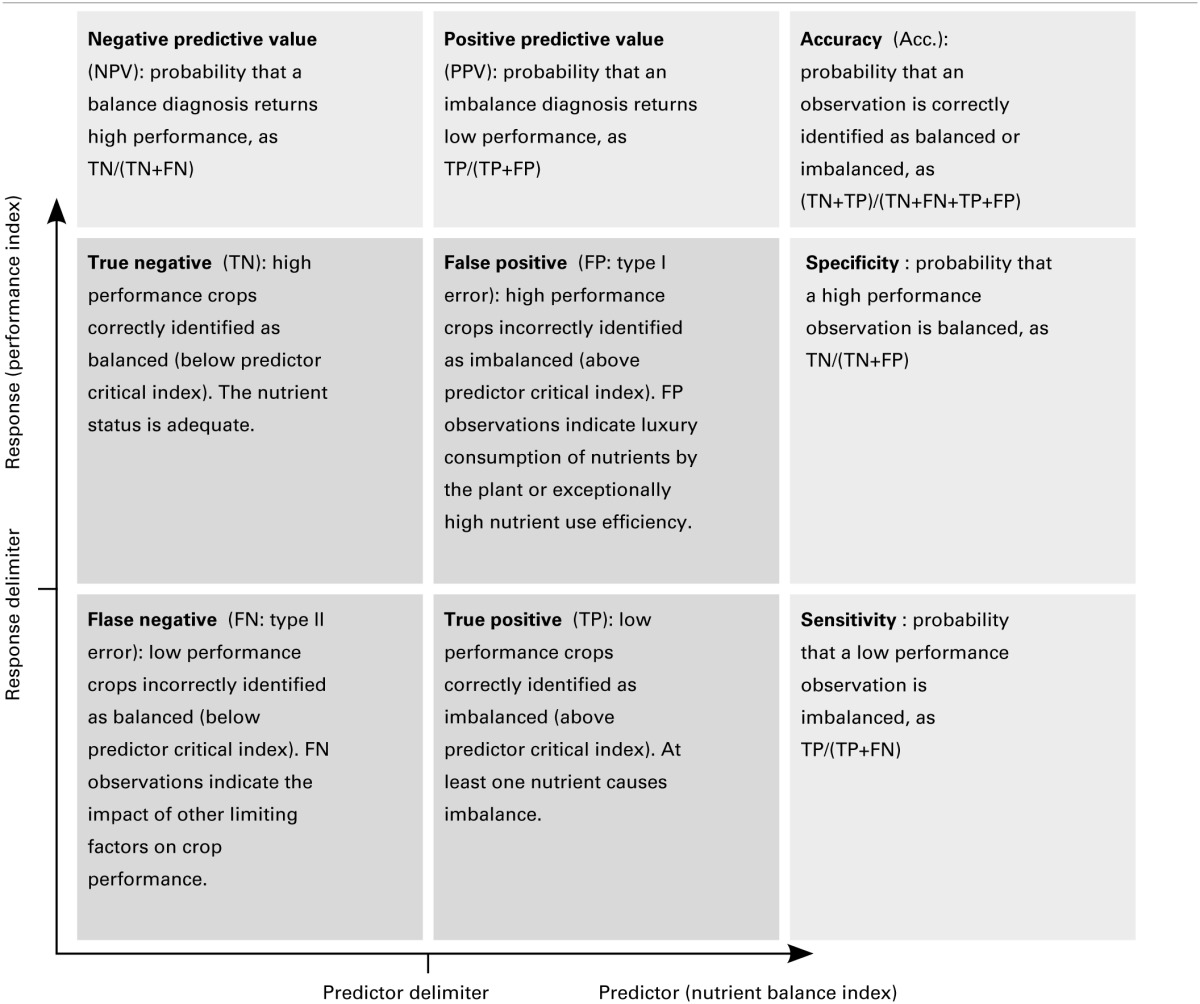
**Term definitions in binary classifications**.

In the Receiver Operating Characteristic (ROC) method, commonly used to measure the performance of clinical tests (Swets, 1988), the selected predictor delimiter corresponds to the best compromise between sensitivity and specificity, i.e., the maximal Youden's *J* index (sensitivity + specificity – 1) (Youden, 1950). The area under the sensitivity vs. specificity curve (AUC) can be used as an accuracy index for the classification (Swets, 1988).

Because crop yield (response) is a continuous variable rather than a binary variable as is the case in most clinical tests, a procedure is needed to optimize the response delimiter, as developed in the Material and methods section. In survey datasets, true negative (TN) specimens represent the reference population. Because the Mahalanobis distance from TNs (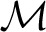
_TN_) is used as predictor, an iterative procedure is needed, as follows. For a given response (crop yield) delimiter, the predictor is initiated using high-yielders as reference specimens for computing 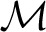
_HY_. Thereafter, a predictor delimiter is selected and its barycenter and co-variance are computed among newly delineated TN specimens in order to compute 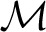
_TN_. The 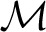
_TN_ is iterated until two iterations classifies observations identically.

## Materials and methods

### Data set

We collected data in 93 “Palmer.” 63 “Tommy,” 14 “Espada,” and 5 “Haden” mango orchards planted between 1983 and 2005 on Oxisols and Ultisols near Jabotocabal in the state of São Paulo, Brazil, for a total of 175 orchards. At the end of July during flowering, leaves were collected from the middle tier of annual growth. Foliar N was determined by micro-Kjeldahl. The S, P, K, Ca, Mg, Zn, Cu, Mn, Fe, and B foliar concentrations were determined by IPC-OES after digestion in a mixture of nitric and perchloric acids (Jones and Case, 1990). Fruits were harvested from five trees randomly selected in each orchard, and averaged as kg tree^−1^.

Phenotypic plasticity of plant ionome may be driven by nutrient supply of soils and fertilization practices. Fertilization practices were assumed to be standard across orchards and only soil properties were measured. Soils were sampled after harvest at four locations per tree in the 0–20 cm layers, then composited per 5-tree experimental unit. Soil samples were air dried and analyzed for pH in 0.01 M CaCl_2_, organic matter content, P, K, Ca, Mg and (H + Al), and micro-nutrients using official Brazilian methods (Raij et al., 1987). Exchangeable acidity (H + Al) was determined by the SMP pH buffer method and the equation of (Quaggio et al., 1997) to convert buffer pH to mmol_*c*_ (H + Al) dm^−3^ as follows:

(6) $$\left(\text{H+Al}\right)=10\text{exp}\left(7.76+1.053pH_{\text{SMP}}\right),R^{2}=0.98$$

### Classification of nutrient balances

The Mahalanobis distance from the median of the TN specimens was used as predictor. The response (productivity criteria) delimiter returning the largest area under ROC curve (AUC) was selected. For statistical validity, the delimiters associated to maximum AUC should also include sufficient data classified in the reference population (TN). Because the amount of data was relatively small, we retained a minimum of 20 observations in the TN quadrant.

### Statistical analysis

Statistical computations were conducted in the R statistical environment (R Development Core Team, 2013) using the R “compositions” package (van den Boogaart et al., 2013). Outliers among *ilrs* were discarded at the 0.01 level using the R “mvoutlier” package (Filzmoser and Gschwandtner, 2013). We compared *ilr* coordinates of ionomes using Tukey's test at a 0.05 significance level. Variances of balances were compared using Bartlett's test and their mean were compared using analysis of variance (*p* = 0.05). Because tests are multivariate and plant data sets contain extreme values, a robust method based on the median is needed to compute multivariate distances (Filzmoser et al., 2009). The Moore-Penrose pseudo-inversion was used to avoid singularities in the inversion of the covariance matrix needed for computations of Mahalanobis distances (Prekopcsák and Lemire, 2012).

## Results

### Varietal ionomes

**Figure 1:**
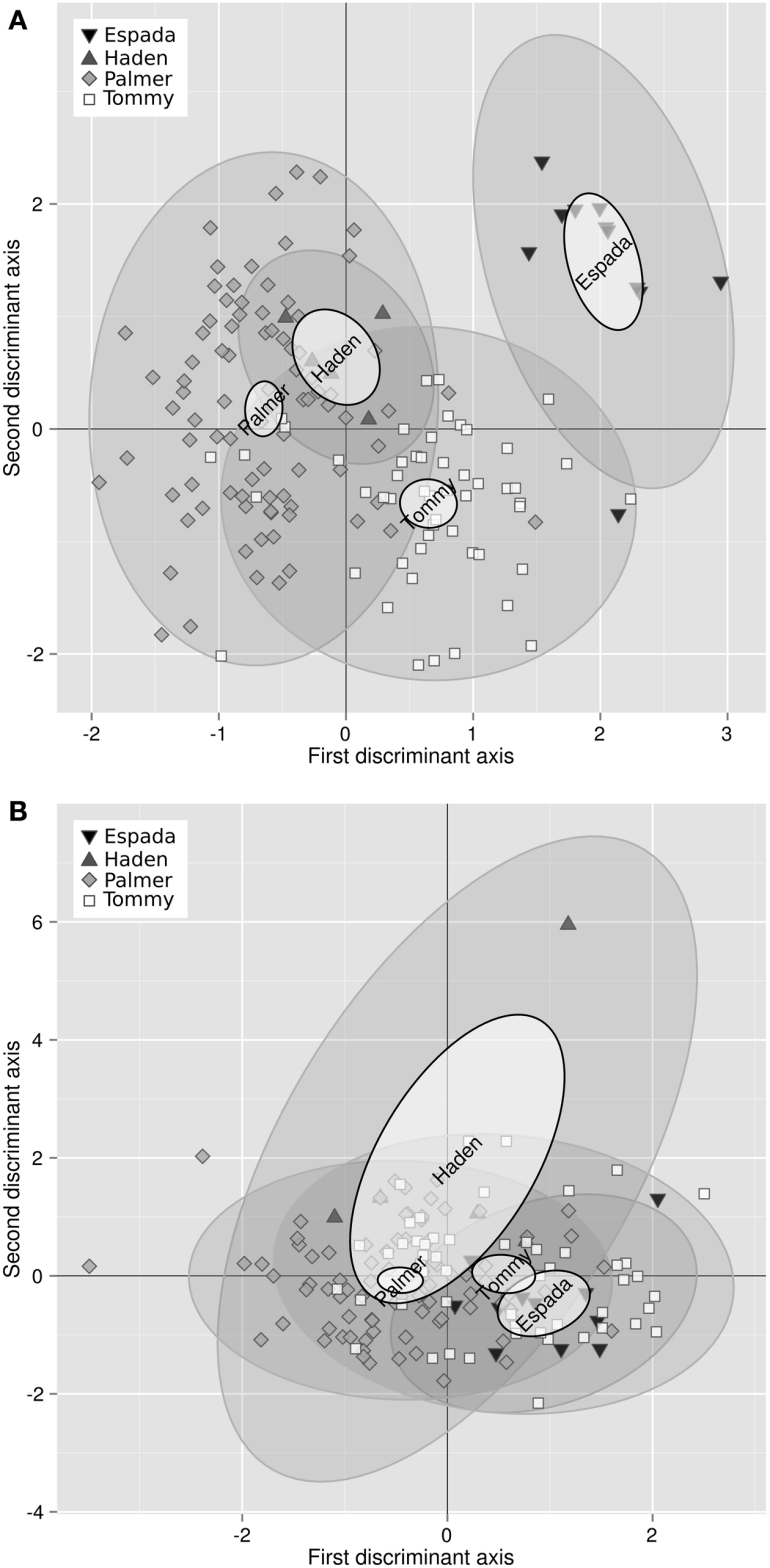
**(A)** Discriminant analysis of the ionomes of four varieties and **(B)** their soil properties (right) in mango orchards in the state of São Paulo, Brazil: “Palmer” (93 obs.), “Tommy” (63 obs.), “Espada” (14 obs.) and Haden (5 obs.). Large semitransparent ellipses that enclose swarms of data points represent regions that include 95% of the theoretical distribution of canonical scores for each species. Smaller plain white ellipses represent confidence regions about means of canonical scores at 95% confidence level.

Bartlett test showed that the variance of 3 of the 11 balances differed among varieties, i.e., [Fv | nutrients], [B | S,N,P,K,Ca,Mg] and [N | S]. Analysis of variance showed that 8 of the 11 balance means differed among varieties, with the exception of [B | S,N,P,K,Ca,Mg] (barely interpretable due to heterogeneous variance), [Mn | Cu,Zn] and [Zn | Cu]. The discriminant scores mapped the differences between ionomes of “Palmer,” “Tommy,” “Espada,” and “Haden” (Figure 1). The plant and soil DA maps showed that ionomes differed significantly between varieties. However, the swarms of foliar ionomes did not overlap among varieties while the swarms of soil nutrients in “Palmer” and “Haden” orchards, on the one hand, and “Tommy” and “Espada,” on the other, overlapped, therefore indicating genotypic dominance over soil nutrient supply. Nevertheless, because the amount of data was limited, the ROC partitioning was performed across varieties to provide a numerical example.

### Binary classification

**Figure 2:**
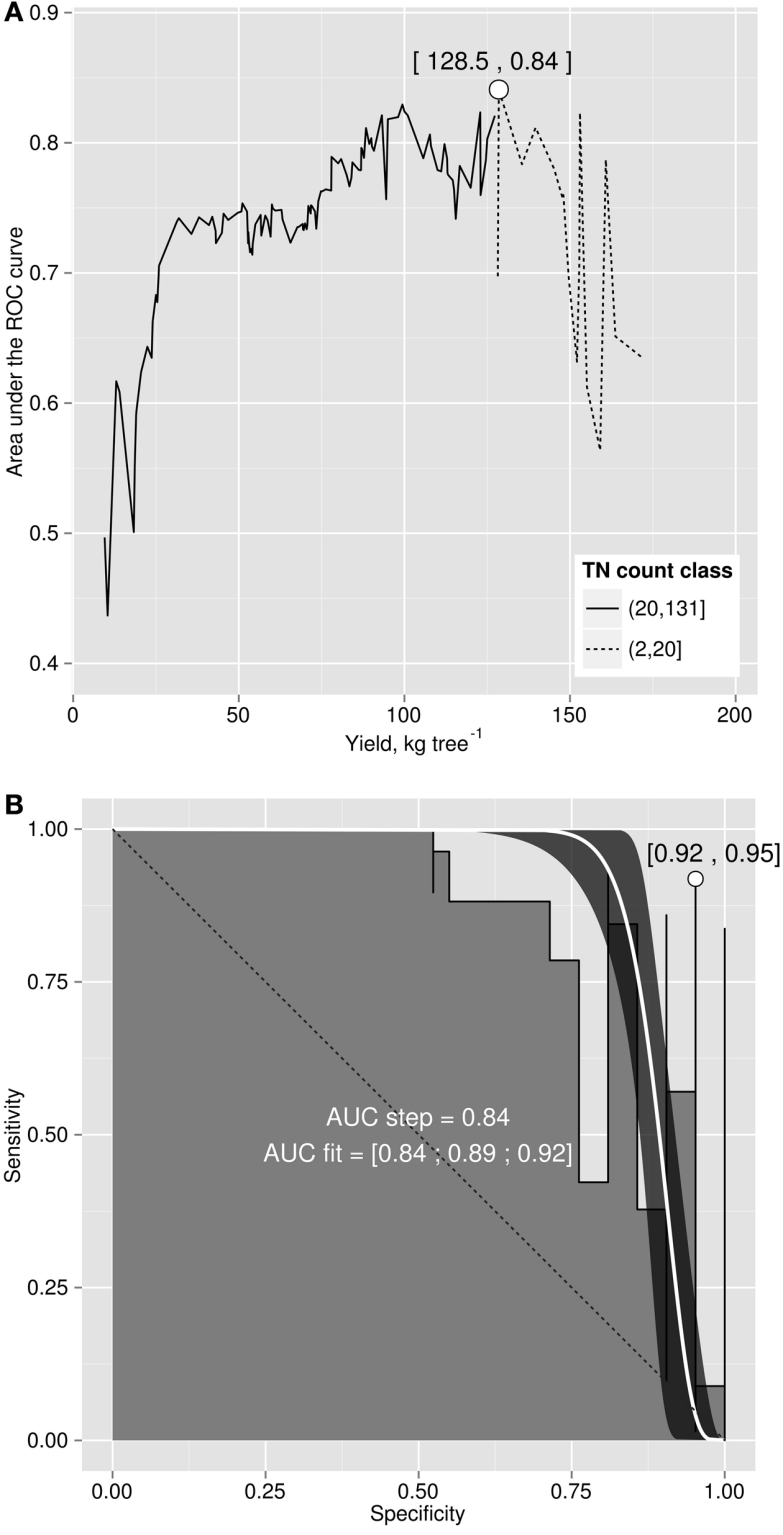
**(A)** area under the ROC curve versus cut-off yield and **(B)** ROC curve for yield cut-off of 128.5 kg fruit tree^−1^.

**Figure 3:**
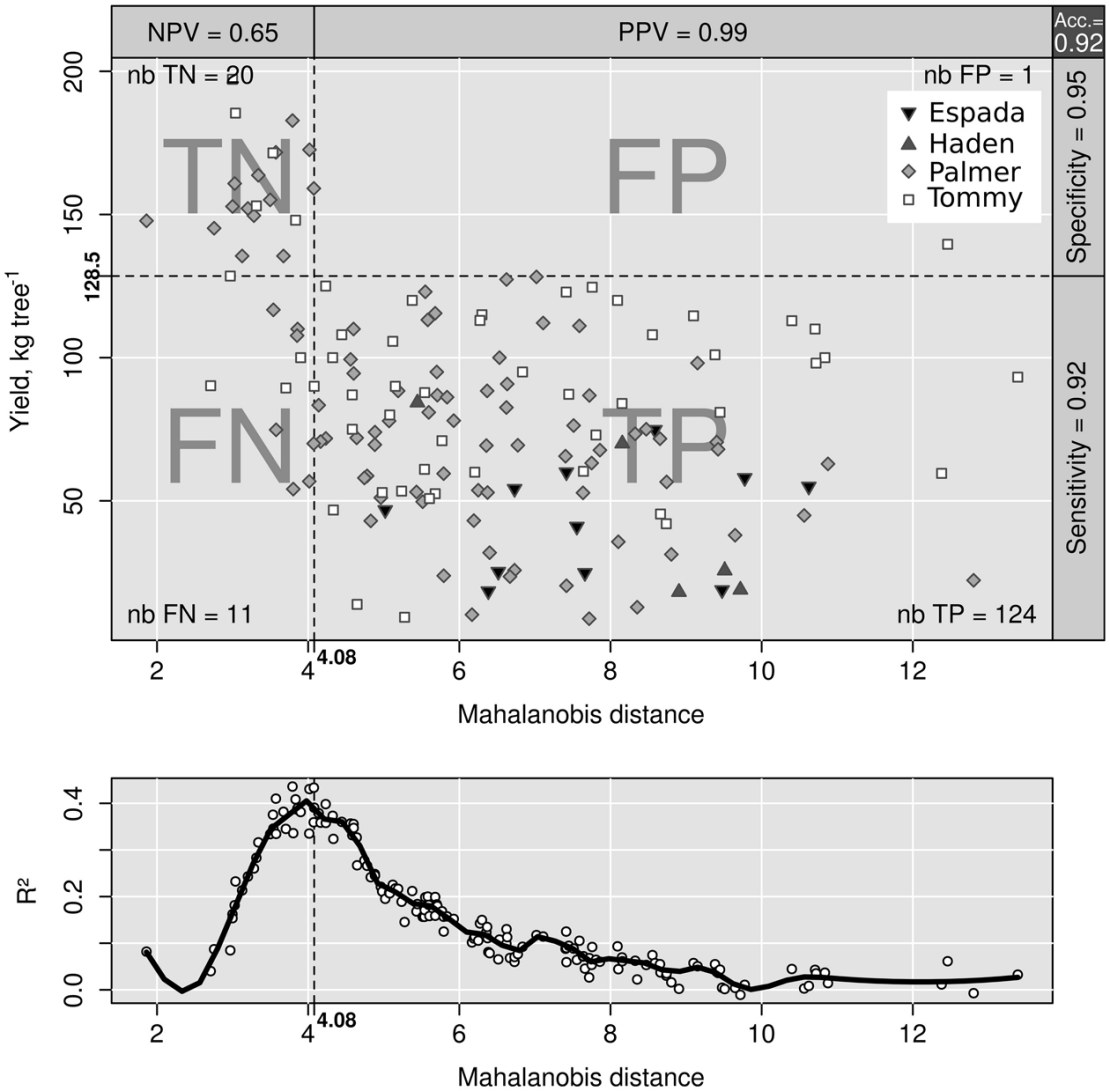
**Binary classification of data with indexes (top).** Class sum of squares (bottom).

The area under the ROC curve (AUC), computed by summing rectangles under the step curve, reached a peak at 0.84 (Figure 2B), a value comparable to the AUC for fairly informative tests (0.80–0.98) in medical sciences (Swets, 1988). The ROC curve did not show a monotonic decrease of sensitivity as specificity increased, as usually observed in ROC diagnoses due to the re-sampling of the TN specimens (see methodology section), which is generally not needed in conventional clinical studies. The AUC computed across the fitted binormal model (Hanley, 1988) returned a value of 0.89, ranging between 0.84 and 0.92, with a confidence level of 95% (Figure 2B). The response delimiter corresponding to the AUC peak was 128.5 kg fresh fruit tree^−1^ (Figure 2A). The optimal compromise between specificity and sensitivity for the optimal response delimiter was found at a specificity of 0.95 and a sensitivity of 0.92, corresponding to a predictor threshold (Mahalanobis distance) of 4.08 (Figure 2B). Results of the binary classification are presented in Figure 3, where the two optimal delimiters classified 20 observations in the TN quadrants. The semi-transparent ellipse enclosed 95% of the theoretical distribution of all observations. The TN group was essentially constituted of “Tommy” (6) and “Palmer” (14) orchards. All “Espada” and “Haden” orchards were classified as TP. The large majority (accuracy = 92%) of specimens were correctly diagnosed by the Mahalanobis distance predictor. Almost all specimens declared imbalanced yielded less than cut-off yield value (PPV = 99%). On the other hand, nearly two thirds (NPV = 65%) of balanced specimens yielded more than cut-off yield value. Median *ilr* values of TN specimens as well as the covariance matrix used to measure Mahalanobis distances are presented in Tables 3 and 4.

**Table 3:** **Confidence intervals of *ilr* values (±*t*_0.025_$\sqrt{s^{2}/n}$) for true negative (TN) specimens (*n* = 20) in the Brazilian mango data set (LL, lower limit; UL, upper limit)**.

	**LL**	**Median**	**UL**
[Fv | S,N,P,K,Ca,Mg,B,Cu,Zn,Mn,Fe]	−7.153	−7.086	−7.000
[Cu,Zn,Mn,Fe | S,N,P,K,Ca,Mg,B]	5.394	5.518	5.859
[B | S,N,P,K,Ca,Mg]	4.454	4.639	4.720
[K,Ca,Mg | S,N,P]	−1.312	−1.205	−1.170
[P | S,N]	1.227	1.300	1.338
[N | S]	−1.652	−1.628	−1.551
[Ca,Mg | K]	0.248	0.345	0.407
[Mg | Ca]	1.563	1.598	1.682
[Fe | Cu,Zn,Mn]	−0.162	−0.005	0.095
[Mn | Cu,Zn]	−2.554	−2.374	−2.131
[Zn | Cu]	−0.148	0.218	0.539

**Table 4:** **Covariance matrix (excluding outliers) of TN specimens of mango observations in the Brazilian data set used to compute the Mahalanobis distance**.

	**ilr1**	**ilr2**	**ilr3**	**ilr4**	**ilr5**	**ilr6**	**ilr7**	**ilr8**	**ilr9**	**ilr10**	**ilr11**
ilr1	0.0267										
ilr2	−0.0653	0.2467									
ilr3	−0.0289	0.0403	0.0808								
ilr4	0.0158	−0.0299	−0.0173	0.0233							
ilr5	0.0068	−0.0344	0.0024	−0.0008	0.0140						
ilr6	0.0076	−0.0233	−0.0025	0.0009	−0.0003	0.0117					
ilr7	0.0053	0.0076	0.0000	0.0109	−0.0092	0.0038	0.0288				
ilr8	−0.0032	0.0003	0.0132	−0.0082	0.0024	0.0039	−0.0039	0.0161			
ilr9	0.0203	−0.0612	−0.0388	0.0196	0.0056	0.0024	0.0003	−0.0106	0.0757		
ilr10	0.0507	−0.1155	−0.0809	0.0330	−0.0048	0.0266	0.0219	−0.0091	0.0617	0.2040	
ilr11	0.0619	−0.2338	−0.0614	0.0111	0.0423	0.0156	−0.0330	−0.0254	0.0626	0.1599	0.5378

### Nutrient balance comparisons between TN and TP specimens

**Figure 4:**
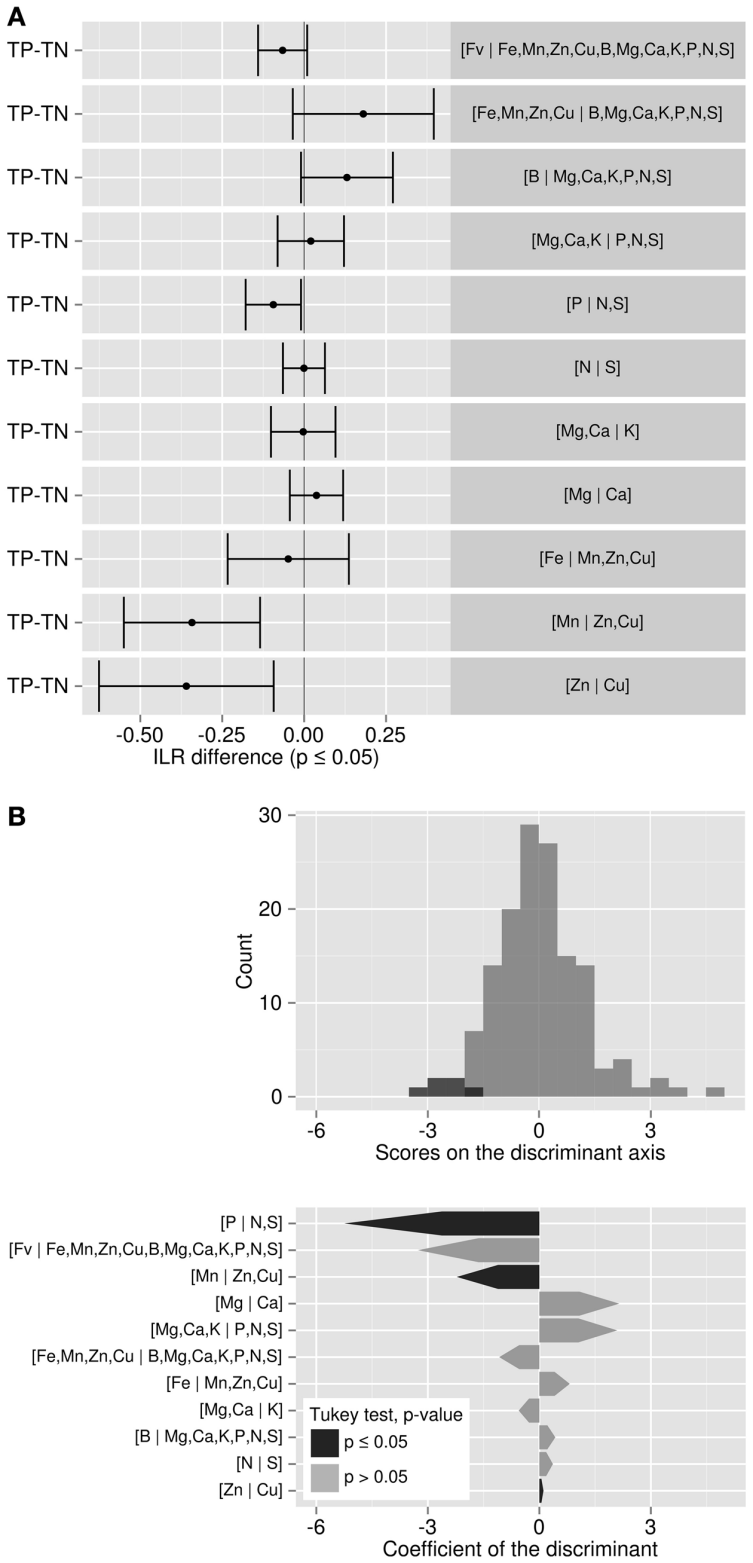
**(A)** Tukey test of *ilr* differences between TP and TN specimens: where balance difference is negative, components on the left side of the balance load more than those on the right side for TP specimens. **(B)** Discriminant analysis between TP and TN specimens.

Tukey's test allowed detecting in which balance significant differences occurred between TN and TP specimens (Figure 4A). The most significantly different balance was [Mn | Cu,Zn] (*p* < 0.01). A negative (TP–TN) value means that TN's balance was higher than TP's. For the [Mn | Cu,Zn] balance, the TN specimens tended to be characterized by greater load in the plus (+) group than the minus (−) group compared to TP. In this case, Cu and Zn loaded more than Mn in the TN ionomes. There was a significant trend for TN specimens to accumulate more Cu relatively to Zn, as shown by the negative [Zn | Cu] balance difference (TP–TN) (*p* < 0.01). However, the [P | N,S] balance was the most discriminant balance between TN and TP specimens (Figure 4B): compared to TP specimens, TN specimens accumulated significantly more S and N than P (i.e., TN had a higher [P | N,S] balance) (*p* < 0.05). There was no significant difference between other balances.

### Pan balance diagram

**Figure 5:**
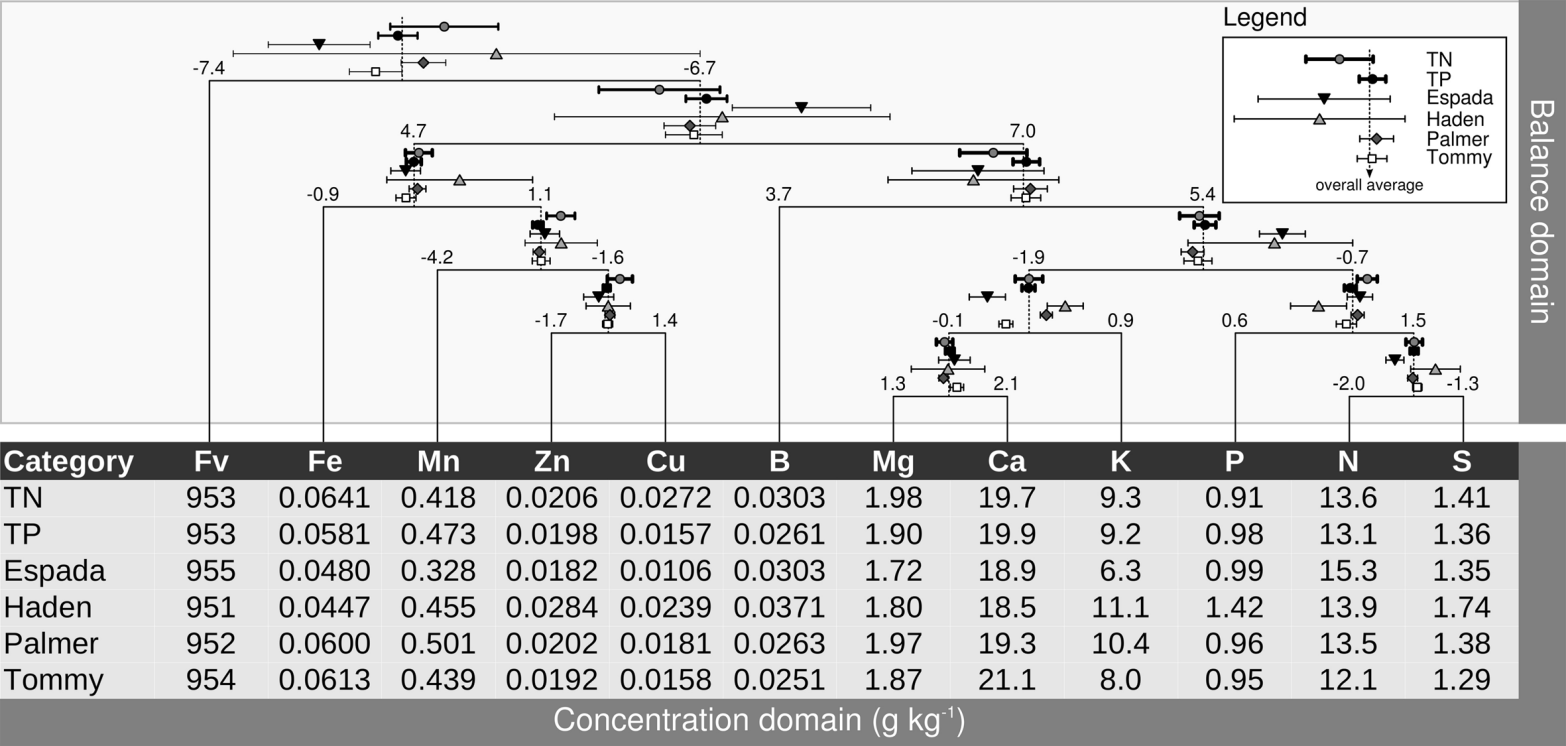
**Pan balance design illustrating nutrient equilibrium in foliar tissues of mango varieties.** Concentrations in weighing pans are back-transformed *ilr* means. TN, true negative specimens; TP, true positive specimens.

Balances can be represented metaphorically using a stand-alone mobile diagram with fulcrums and weighing pans, where nutrient concentrations in buckets impact directly on nutrient balances at fulcrums upon change. Figure 5 presents a balance dendrogram derived from SBP with overall average *ilr* values at fulcrums, and 0.05 univariate confidence intervals for TN specimens, TP specimens, and each variety.

The *ilr* values at fulcrums are used for diagnostic purposes, while the *ilr* values back-transformed to familiar concentration units are laid down in buckets to provide an appreciation of balances in terms of relative shortage, adequacy, luxury consumption or excess of contributing nutrients. Differences between TN and TP specimens can be observed in the pan balance diagram. There were marked differences between the TNs and TPs in Cu concentration, apparently misbalancing significantly [Mn | Zn,Cu] and [Zn | Cu]. Although P shortage seemed to be small in TP specimens, it contributed to misbalance [P | N,S] in TP specimens.

The [Fe | Mn,Zn,Cu] misbalance of “Haden” specimens, although not significant due to too few observations (5), is attributable to a balance driven positively by relatively low Fe and high Cu levels. The “Espada” [Fe | Mn,Zn,Cu] balance was similar to that of “Tommy” and “Palmer.” Even though “Espada” showed relatively low Fe, Mn, Zn and Cu levels, this apparent shortage of nutrients was properly balanced. However, those low concentrations were misbalanced with other nutrients because the [Fe,Mn,Zn,Zn,Cu | B,Mg,Ca,K,P,N,S] fulcrum leaned to the right (positive side). Also, low Mg and low Ca in “Espada” specimens misbalanced [Mg,Ca | K] while maintaining [Mg | Ca] properly balanced. The [N | S] balance of “Espada” significantly leaned to the left due to relative N excess. The “Espada” [Fv | Nutrients] balance departed significantly from TNs on the negative side, indicating overall relative nutrient shortage. Although balances related to micronutrients were within TN range, “Tommy” was largely misbalanced by relative K shortage and somehow by relative Mg shortage and, apparently, relative Ca excess. The addition of K and Mg fertilizers to “Tommy” could likely re-establish the [Fv | Nutrients] balance. Most balances of “Palmer” were within TN balance ranges, except for [Mg,Ca | K], mostly due to relative K excess. This balance could be likely re-established by adding Mg and Ca or reducing or omitting K additions, with some risk of misbalancing other balances.

### Biases

**Figure 6:**
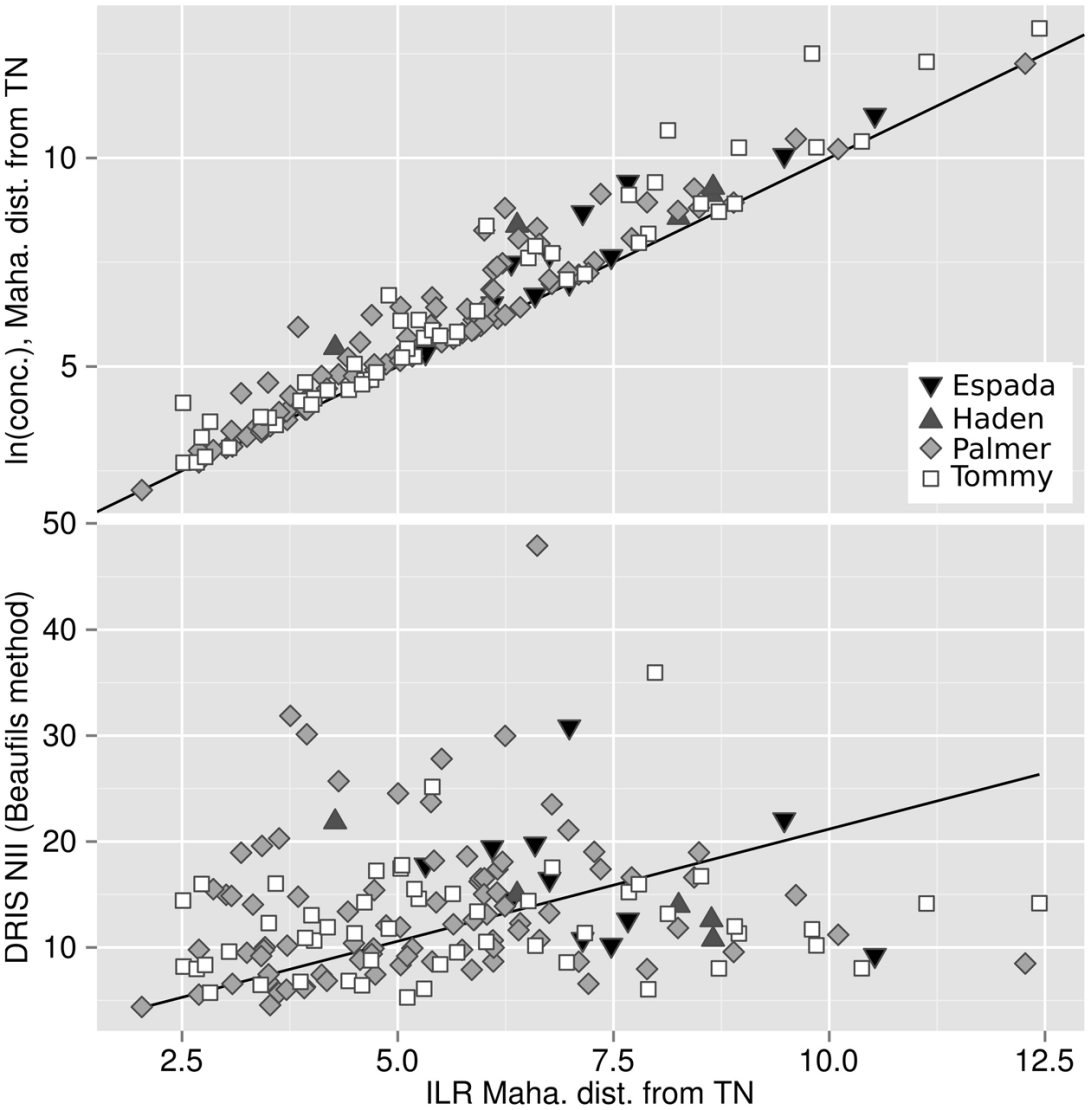
**Bias measured by discrepancy between the Mahalanobis distance from the TN population across the isometric log ratios (x-axis) and (top) the Mahalanobis distance from the TN population across the natural log of concentrations and (bottom) the DRIS nutrient imbalance index**.

The Mahalanobis distance from TN specimens based on the natural log of concentrations, as well as the DRIS nutrient imbalance indexes using TN specimens as standards, were compared to the Mahalanobis distance from TN specimens based on unbiased *ilr* balances in Figure 6. The bias of the approach can be appreciated quantitatively by the importance of the departure from the 1:1 line on the plot where concentrations are log-transformed (*R*^2^ = 0.017) and by the importance of the residuals where concentrations are computed as DRIS NII (*R*^2^ = 0.48). Both approaches (log of concentrations and DRIS) produced noisy diagnoses, possibly leading to conflicting interpretations.

## Discussion

### Mango ionomes

Variation in ionomes could be interpreted only partly as genotypic effect because phenotypic plasticity can also be driven by differential nutrient supplies. Phenotypic plasticity is a phenomenon typical of domesticated species that are most often bred for high productivity under relatively luxurious environments (Chapin III, 1980, 1988). Because mango varieties were developed almost essentially (90%) from the germplasm of *Mangifera indica* (Mukherjee, 1963), nutrient management of mango orchards are generally thought to be related to yield potential at species rather than variety level. We found that genotypic variation in nutrient balance could also be addressed in future research when introducing new mango varieties in commercial orchards, as shown especially by the differential ionomes of “Espada” compared to “Tommy” despite similar nutrient supply.

There is often a large number of misclassified false negative specimens in fruit crop survey datasets not only due to small climatic variations and natural or pathological changes occurring in trees, but also to biennal fruit bearing habits alternating between “on-year” and “off-year” (Monselise and Goldschmidt, 1982). However, proper pruning of the mango tree presumably limited the effect of alternate bearing on fruit yield in the surveyed orchards.

### Biases

The positive shift of the Mahalanobis distances based on natural log transformations from the one based on *ilr* balances (Figure 6) is due to the general overestimation of distances computed across untransformed or log-transformed compositions (Lovell et al., 2011). Filzmoser et al. (2009) argued that a log ratio transformation is similar to a log transformation only when a large filling value is used as denominator, because $\underset{x_{fv}\to 1}{\lim}\left(alr(x)-ln(x)\right)=0$. We showed that, for mango ionomes, biases using log-transformed concentration data distorted the multivariate diagnosis despite large filling values. This result indicates that log ratio transformations are preferable to log transformations to avoid biases when handling plant nutrient data that physiologically interact with each other.

### Nutrient rebalancing

When using ratios for diagnostic purposes, it is impossible to figure out whether a nutrient level is too high, adequate or too low: this appeared to be a definite weakness for nutrient ratio interpretation (Walworth and Sumner, 1987; Wilkinson, 2000; Marschner, 2011). However, the pan balance approach connects nutrient balances and concentrations within a physiologically sound, coherent, and statistically unbiased model, where concentrations can assist in appreciating the results of statistical analyses performed on isometric log ratios. Using *ilrs* at fulcrums for unbiased diagnosis and nutrient concentrations in buckets to provide an appreciation of the results as relative shortage, adequacy or excess compared to TN barycentres, plant diagnosticians are informed at a glance on how concentration levels impact on nutrient balances. We thus suggest a paradigm shift from the traditionally combined and potentially conflicting CNCR-DRIS diagnoses of nutrient status to the stand-alone pan balance metaphor illustrated by a mobile-and fulcrums setup with buckets loaded with nutrient concentrations.

The analyst should keep in mind that nutrient deficiency, sufficiency or excess of any nutrient can only be diagnosed in relation to other nutrients. Only balances can be tested statistically without bias. The weighing pans facilitate interpreting the balances correctly. For example, the lower the [Mg | Ca] balance in TN specimens can be appreciated as a combination of lower Ca and higher Mg concentrations compared to TP specimens and this is ascertained looking at concentration values associated with the corresponding TN nutrient loads in weighing pans. Corrective measures involving one element may impact on all balances connected to it. This is why the effect of corrective measures on such complex system should be confirmed by experimentation and monitoring in many cases.

The primary misbalance in mango orchards appeared to be [P | N,S] and [Mn | Cu,Zn]. The [P | N,S] is related to the balance between energy and protein synthesis (Loladze and Elser, 2011), while the [Mn | Cu,Zn] balance depends on soil properties and fungicide applications. There was a narrow range of balances for [P | N,S], indicating that fertilization should be conducted carefully to avoid nutrient misbalance. The relations between N, P, and S, as well as Mn, Cu, and Zn, could be appreciated in the concentration domain to identify the buckets that are under- (relative deficiency) or over-loaded (relative excess) with nutrients.

## Conclusion

Using a Brazilian mango data set of crop productivity and plant and soil compositions, we addressed two typical problems when diagnosing the mineral nutrition of fruit crops: (1) genotype effect versus phenotypic plasticity and (2) double-biased diagnosis with CNCR and DRIS conducted separately versus a coherent stand-alone balance-concentration setup. Because ionomes are made of compositional data, former diagnostic tools developed according to the “Law of minimum” and illustrated by Liebig's barrel in agronomic studies should obviously be replaced by more modern theories and numerical tools. The pan balance metaphoric representation of *ilr* variables is a novel model that integrates statistical diagnosis of balances and qualitative evaluation of nutrient concentrations into a unified and coherent diagnosis that avoids conflicting interpretation of nutrient concentrations and ratios when diagnosed separately.

In the mobile device, the way the SBP is designed impacts on the diagnosis, because confidence ranges of the reference group (TN) are not multivariate, but a collection of interpretable univariate ranges. However, the Mahalanobis distance, being independent of the SBP, is a robust indicator of nutrient balance. Future research should focus on fertilizer trials moving imbalanced nutrient profiles below the critical Mahalanobis distance from a reference group. The choice of balances in this paper was based on prior knowledge that could be ascertained by multivariate analysis of large and diverse data sets. This would require analyzing metafiles across several species, genotypes, soil types, irrigated or rainfed production systems and sampling periods. The ROC procedure developed in this paper could be instrumental in comparing the performance of tissue and soil testing as tools of nutrient management in cropping systems.

## Author contributions

- *Serge-Étienne Parent* analyzed data, developed statistical tools, took care of numerical aspects and co-wrote the paper.
- *Léon Etienne Parent* took care of the theoretical background in plant nutrition, analyzed results, supervised data analysis and co-wrote the paper.
- *Danilo Eduardo Rozane* collected and analysed samples.
- *William Natale* supervised field and laboratory campaigns and provided data.

### Conflict of interest statement

The authors declare that the research was conducted in the absence of any commercial or financial relationships that could be construed as a potential conflict of interest.

## Abbreviations

Acc.: accuracy
CND: Compositional Nutrient Diagnosis
DRIS: Diagnosis and Recommendation Integrated System
FN: false negative
FP: false positive
NPV: negative predictive value
PPV: positive predictive value
ROC: receiving operating characteristic
TN: true negative
TP: true positive.
